# Suppression of Pathological Allergen-Specific B Cells by Protein-Engineered Molecules in a Mouse Model of Chronic House Dust Mite Allergy

**DOI:** 10.3390/ijms252413661

**Published:** 2024-12-20

**Authors:** Nikola Ralchev, Silviya Bradyanova, Nikola Kerekov, Andrey Tchorbanov, Nikolina Mihaylova

**Affiliations:** Department of Immunology, Institute of Microbiology, Bulgarian Academy of Sciences, 1113 Sofia, Bulgaria; nikola_ralchev@microbio.bas.bg (N.R.); silvybradyanova@microbio.bas.bg (S.B.); nikolakerekov@gmail.com (N.K.); tchorban@microbio.bas.bg (A.T.)

**Keywords:** chronic house dust mite allergy model, chimeric molecules, FcγRIIb receptors

## Abstract

Der p1 is one of the major allergens causing house dust mite (HDM) allergy. Pathological Der p1-specific B cells play a key role in allergic inflammation as producers of allergen-specific antibodies. Crosslinking the inhibitory FcγRIIb with the B cell receptor triggers a high-affinity suppressive signal in B cells. Selective elimination of allergen-specific cells could potentially be achieved by administering chimeric molecules that combine, through protein engineering, the FcγRIIb-targeting monoclonal 2.4G2 antibody with the epitope-carrying Dp52–71 peptides from Der p1. We tested this hypothesis, in a chronic mouse model of HDM allergy induced in BalB/c mice, using FACS and ELISA assays, along with histopathological and correlational analyses. Dp52–71chimera treatment of HDM-challenged mice led to a decrease in serum anti-HDM IgG1 antibodies, a reduction in BALF β-hexosaminidase levels, a lowered number of SiglecF^high^ CD11c^low^ eosinophils, and an improved lung PAS score. Furthermore, we observed overexpression of FcγRIIb on the surface of CD19 cells in the lungs of HDM-challenged animals, which negatively correlated with the levels of lung alveolar macrophages, neutrophils, and BALF IL-13. Taken together, these results suggest that the use of FcγRIIb overexpression, combined with the expansion of chimeric protein technology to include more epitopes, could improve the outcome of inflammation.

## 1. Introduction

House dust mites (HDMs) are widely spread and are recognized as one of the primary sources of indoor allergens. Approximately 1–2% of the world’s population is sensitized to HDM allergens [1]. HDM allergy is related to various conditions, including atopic dermatitis and allergic rhinitis. In addition, approximately 50% of all individuals with asthma are sensitized to these allergens [2,3].

The management of allergies typically involves allergen avoidance and pharmacological treatments aimed at alleviating disease symptoms. However, the symptomatic relief of HDM allergy ends once the treatment is discontinued or the patient encounters an allergen [4]. Furthermore, many of the current pharmacological agents for allergic rhinitis or asthma have not been investigated specifically regarding HDM sensitization, or have resulted in poor to moderate symptom management in some patients, as well as having the capability to cause significant side effects [4,5]. Allergen immunotherapy (AIT) is the only established treatment that directly targets the underlying allergic mechanisms and provides long-term benefits. Nevertheless, AIT requires lengthy protocols, usually lasting 3–4 years, and there are uncertainties in terms of its cost-effectiveness and long-term efficacy. These factors contribute to the fact that AIT is used in less than 10% of allergic rhinitis and asthma cases. Furthermore, numerous studies are currently investigating novel routes of administration aiming to enhance therapeutic efficacy [6,7,8,9]. Therefore, innovative treatment approaches that can influence the long-term management of HDM allergies are necessary. Allergen-specific B cells with an IgE class-switched isotype play a key role in the pathological mechanism of allergic diseases. Secreted allergen-specific IgE antibodies mediate allergic response through binding to high-affinity FcεRI receptors on the surface of mast cells and basophils. A subsequent challenge with allergens results in the release of bioactive mediators from these cells, leading to allergic symptoms [10,11]. Furthermore, the administration of the monoclonal anti-IgE antibody omalizumab reduces disease symptoms in allergic patients [12,13]. The important role of IgE antibodies outlines allergen-specific B cells as a logical target for the treatment of HDM allergy. A novel therapeutic approach leading to the selective elimination of allergen-specific B cells can be considered as a potential therapy, with a disease-modifying effect without the requirement of lengthy and difficult-to-adhere-to protocols.

The house dust mite *Dermatophagoides pteronyssinus* (Dpt) is an important allergen source causing HDM allergic disease [14]. Der p1 is the immunodominant and most abundant allergen in *Dpt* cultures, with more than 80% of HDM-allergic patients developing IgE antibodies against it. In addition, 50–60% of all anti-HDM IgE antibodies are specific to Der p1 and Der p2 allergens [15,16]. Prior studies have identified four Der p1 B cell epitopes, and their IgE-dependent biological activities have subsequently been analyzed [17,18,19]. Two epitopes, p52–71 and p117–133, have emerged as inducing more intense and frequent responses in FcεR-expressing cells from HDM-sensitized patients [20]. Peptide p52–71 was employed in our previous study to target pathological human Der p1-specific B cells using protein-engineered chimeric molecules. These chimeras consist of monoclonal antibodies against inhibitory human complement receptor 1 (CR1), coupled with selected Der p1 epitope-carrying peptides, to target and suppress the pathological B cells. In vitro experiments have shown that the chimeric molecules reduce the number of anti-HDM IgE antibody-secreting cells [21]. Furthermore, in vivo administration of the chimeric molecules in a humanized Rag2- γc- mouse model of HDM allergy reduced allergen-specific IgE antibodies and overall inflammation in the lungs [22]. While humanized mouse models are a valuable tool for studying pathological donor cells in vivo, they do have limitations, such as differences in reactivity between rodents and humans [23,24,25].

In contrast to human CR1, which is a negative regulator of B cell receptor (BCR)-dependent activation, mouse CR1 exerts a different function in B cells [26]. Instead, mouse FcγRIIb (CD32) receptor crosslinks with BCR by immune complexes to inhibit B cell activation [27]. Furthermore, this co-ligation results in B cell proliferation arrest and subsequent induction of apoptosis [28]. Our previous study demonstrated that we could induce this negative feedback on antibody secretion in lupus-prone mice using a chimeric molecule, which was constructed by combining a monoclonal antibody against FcγRIIb with a DNA mimotope peptide (DWEYSVWLSN). Administration of these chimeric molecules resulted in the selective suppression of disease-associated DNA-specific B cells, a reduction in serum anti-DNA IgG antibody levels, and a delay in lupus disease manifestations in MRL/lpr mice [29].

This study aims to investigate a novel therapeutic approach for the treatment of HDM allergy by targeting Der p1-specific B cells. We hypothesize that administration of a chimeric molecule, composed of a monoclonal 2.4G2 antibody against FcγRIIb coupled with a Der p1-derived peptide (Dp52–71), can selectively eliminate disease-associated allergen-specific B cells in a chronic mouse model of HDM allergy.

## 2. Results

### 2.1. Characterization of the Chimeric Molecules

**Figure 1:**
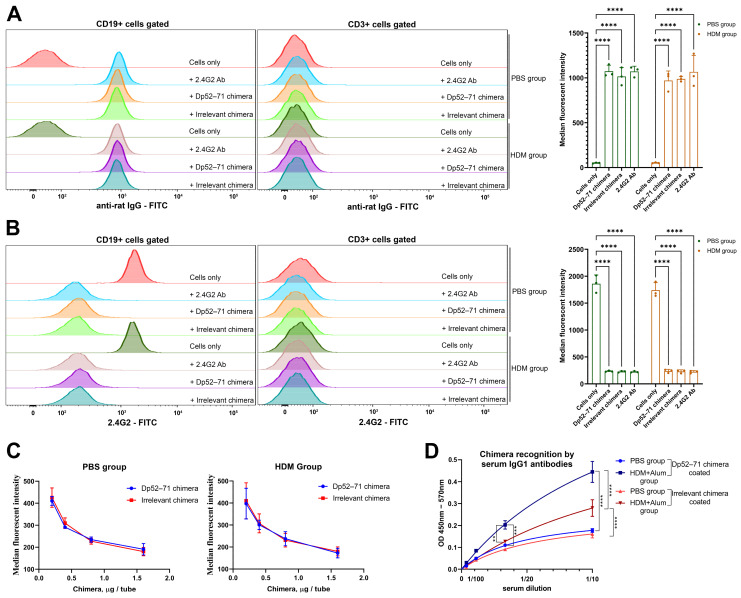
The Dp52–71chimera binds to the FcγRIIb receptor on murine B cells and is recognized by epitope-specific serum IgG1 antibodies. (**A**) Dp52–71 chimera and irrelevant chimera binding on the surface of CD19 and CD3 cells was proved by FACS analysis. Splenocytes from healthy and HDM-challenged mice were incubated with both chimeras and secondary incubated with FITC-conjugated anti-rat IgG (left part). Summarized graph for the median fluorescent intensity (MFI) of anti-rat–FITC antibody fluorescence (right part). (**B**) Dp52–71 chimera competes with commercial 2.4G2-FITC antibody for the same receptor. The same splenocytes were pre-incubated with Dp52–71 chimera, irrelevant chimera, and pure 2.4G2 antibody, and secondary incubated with 2.4G2-FITC antibody (left part). Gated CD19 and CD3 cells were analyzed by FACS (left part). Summarized data of the 2.4G2-FITC binding to the FcγRIIb receptor on B cells (right part). (**C**). Dose-dependent inhibition of the 2.4G2-FITC binding to the FcγRIIb receptor. (**D**). Peptide recognition on the Dp52–71 chimera by serum IgG1 antibodies from HDM + Alum-sensitized mice analyzed by ELISA. Data are represented as mean ± SD of at least 3 mice per group. The differences between the groups were evaluated using a two-way ANOVA following Tukey’s multiple comparisons test; ** *p* < 0.01; *** *p* < 0.001; **** *p* < 0.0001. Data are representative of at least 5 independent experiments.

Dp52–71 and irrelevant chimeras were constructed by coupling rat 2.4G2 monoclonal antibodies with peptide epitopes from Der p1 or irrelevant peptides, respectively. Mass-spectral analysis data indicate that 14–16 peptides are conjugated to each single IgG molecule [30]. The capacity of the chimeric molecules to bind to FcγRIIb receptors was studied by FACS analysis, using splenocytes from healthy and HDM-treated mice. Both the Dp52–71and irrelevant chimeras bound to the surface of CD19 cells with the same intensity as the pure 2.4G2 antibody in PBS- and HDM-challenged mice. No such binding was observed in the CD3 population (Figure 1A).

We further analyzed the capacity of the chimeric molecules to compete against a commercial FITC-conjugated 2.4G2 antibody for binding to the FcγRIIb receptor. Both the chimeras and the pure 2.4G2 antibody showed a similar capacity to engage the FcγRIIb receptor, and effectively prevented binding of the commercial 2.4G2-FITC antibody on CD19 cells (Figure 1B). The observed binding inhibition occurred in a dose-dependent manner (Figure 1C).

The presence of Der p1 peptide epitopes on the Dp52–71 chimera was confirmed by ELISA. Serum IgG1 antibodies from animals sensitized with HDM plus alum recognized the Dp52–71 chimera significantly more than the irrelevant chimera (Figure 1D).

### 2.2. Serum Levels of HDM-Specific Antibodies and Total IgE

**Figure 2:**
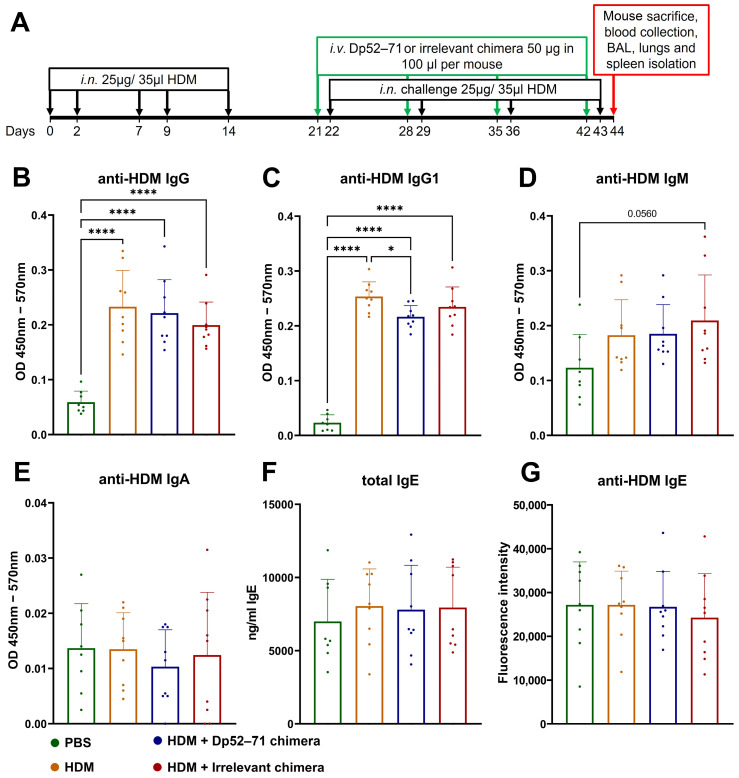
Scheme of the chronic HDM allergy model and treatment schedule (**A**). Serum levels of anti-HDM IgG (**B**), IgG1 (**C**), IgM (**D**), IgA (**E**), IgE (**G**), and total IgE antibodies (**F**) in healthy mice and HDM-challenged mice treated with PBS, Dp52–71 chimera, or irrelevant chimera, measured by ELISA. Data are shown as mean ± SD of 8–9 mice per group. *p* values are calculated using a one-way ANOVA following Tukey’s multiple comparisons test; * *p* < 0.05; **** *p* < 0.0001. Data are representative of at least 3 independent experiments.

A model of chronic HDM allergy was developed to evaluate the effects of chimeric molecules on the onset of allergic inflammation (Figure 2A). Mice were randomized and divided into four groups. One healthy control group was administered i.n. and i.v. PBS (PBS group), while the three HDM-challenged groups were sensitized to i.n. and challenged with HDM extract. The HDM-challenged animals were injected with i.v. with PBS (HDM group), Dp52–71 chimera (HDM + Dp52–71 chimera group), or irrelevant chimera (HDM + irrelevant chimera group).

The effect of chimeric molecules on the levels of allergen-specific antibodies in the mouse sera was assessed by ELISA. HDM-specific IgG and IgG1 antibody levels were elevated in the HDM-challenged groups compared to the PBS group (Figure 2B,C). The levels of anti-HDM IgG1 antibodies were significantly decreased in the group treated with Dp52–71 chimera compared to the HDM group (Figure 2C). This effect was not observed in the mice treated with irrelevant chimera. No significant differences were found in anti-HDM IgG antibody levels between the HDM group and the two chimera-treated groups (Figure 2B).

Serum HDM-specific IgM antibody levels were not significantly increased in the HDM-challenged groups compared to those in the PBS-treated mice. However, such a trend was observed in the irrelevant chimera group, which reached the highest difference (*p* value = 0.056). The serum levels of total IgE (Figure 2F) were not significantly different between the groups, while the levels of HDM-specific IgA antibodies (Figure 2E) did not pass, or barely passed, the limit of detection (LOD), and could be considered negative. The calculated LODs for the ELISA analyses investigating serum anti-HDM antibodies were as follows: IgA-0.008484, IgG1-0.0489, IgG-0.0399, and IgM-0.019653. HDM-specific IgE antibodies were examined by high-sensitivity fluorescence ELISA. No differences were observed between the healthy controls and the HDM-challenged mice (Figure 2G).

### 2.3. Protein Analyses of BALF

**Figure 3:**
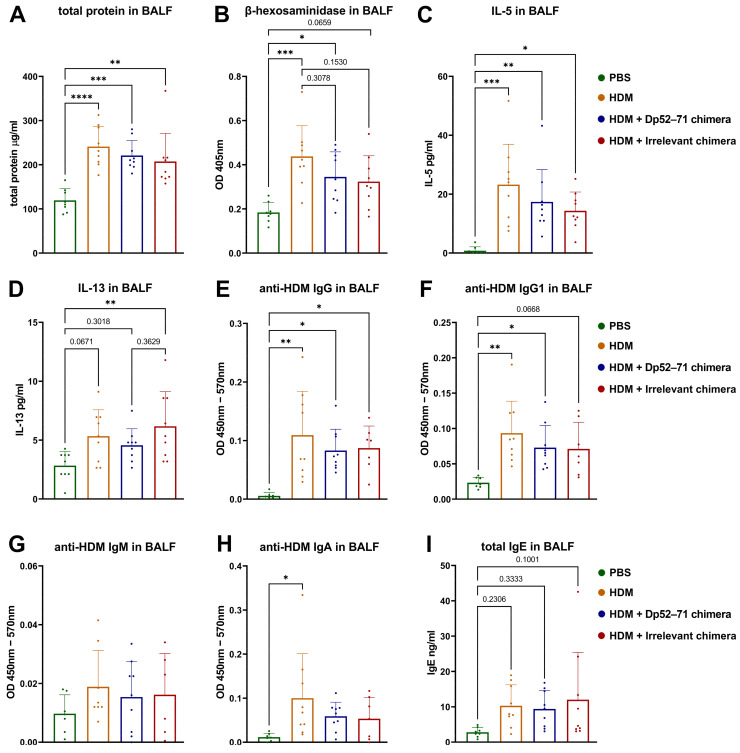
Protein analysis of BAL fluid performed by ELISA. BALF levels of total protein (**A**), β-hexosaminidase activity (**B**), IL-5 (**C**), IL-13 (**D**), HDM-specific IgG (**E**), IgG1 (**F**), IgM (**G**), IgA (**H**), and total IgE (**I**) were investigated in all groups. Data are shown as mean ± SD of 6–9 mice per group. The differences between the groups were evaluated using a one-way ANOVA, following Tukey’s multiple comparisons test; *p* values are indicated on the graphs: * *p* < 0.05; ** *p* < 0.01; *** *p* < 0.001; **** *p* < 0.0001. Data are representative of at least 3 independent experiments.

We investigated several key proteins in BAL fluid to assess the characteristics of the local immune response. Total protein concentration in BALF is used as a marker of vascular permeability [22,31]. Another useful parameter is mast cell degranulation in the lungs, which can be measured using a β-hexosaminidase activity assay in BALF [22,32]. The total protein concentration (Figure 3A) and the enzyme activity (Figure 3B) were increased in all the HDM-challenged groups. A non-significant trend of decreased protein concentration and β-hexosaminidase activity was observed in the HDM-challenged animals treated with Dp52–71 and in the irrelevant chimera group, compared to the HDM group.

The HDM-challenged group showed elevated levels of IL-5 (Figure 3C), anti-HDM IgG (Figure 3E), and IgG1 antibodies (Figure 3F) compared to the healthy mice. Again, a reduction in these parameters was detected in the Dp52–71 and the irrelevant chimera groups compared to the HDM group, but the difference was not statistically significant.

The BALF concentration of IL-13 was higher in the irrelevant chimera group than in the healthy animals, but such a statistically significant difference was not observed in the Dp52–71 chimera group (Figure 3D). Elevated levels of anti-HDM IgA antibodies in BALF were observed in the HDM-challenged group compared to the PBS mice (Figure 3H). No statistically significant differences were observed in HDM-specific IgM (Figure 3G), total IgE (Figure 3I), IL-4, and IL-9 levels between the groups.

### 2.4. Total and Differential Cell Count in BAL Fluid

**Figure 4:**
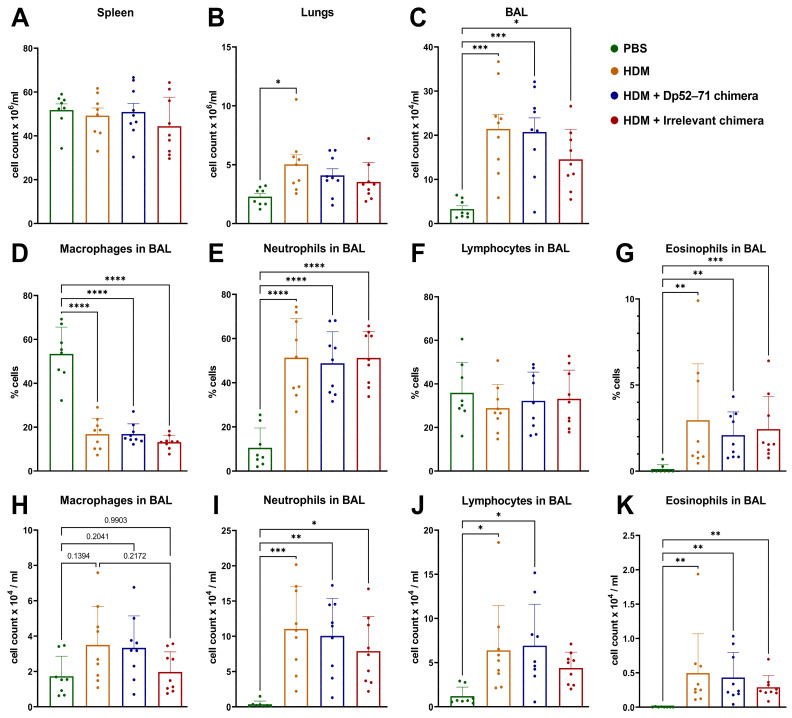
Total cells (per milliliter) isolated from spleen (**A**), lungs (**B**), and BAL (**C**). Differential staining of cells in BAL, defining macrophages (**D**,**H**), neutrophils (**E**,**I**), lymphocytes (**F**,**J**), and eosinophils (**G**,**K**), and expressed as percentages and cell count per ml of recovered BAL liquid. Data are shown as mean ± SD of 8–9 mice per group. The differences between the groups were evaluated using a one-way ANOVA followed by Tukey’s multiple comparisons test, or a Kruskal–Wallis test followed by Dunn’s multiple comparisons test, depending on the normality of the data; *p* values are indicated on the graphs: * *p* < 0.05; ** *p* < 0.01; *** *p* < 0.001; **** *p* < 0.0001.

There was no difference in the number of spleen cells between the groups (Figure 4A), but the total number of isolated cells from the lungs was significantly higher in the HDM-challenged group compared to the PBS-treated animals (Figure 4B). A non-significant decrease in this parameter was found in the Dp52–71 and the irrelevant chimera groups. All the HDM-challenged groups showed increased total BAL cell counts compared to the animals exposed to PBS treatment (Figure 4C). Again, a non-significant trend for a lower number of BAL cells was detected in the irrelevant chimera group (*p* = 0.2686).

The differential BAL cell count revealed a lower percentage of macrophages in all the HDM-exposed groups compared to the PBS-treated mice (Figure 4D). Increased percentages and cell counts of neutrophils (Figure 4E,I), eosinophils (Figure 4G,K), and lymphocytes were detected in the groups challenged with HDM allergens relative to the healthy mice. There were no statistically significant differences in these parameters between the HDM-challenged groups.

### 2.5. Phenotyping of Immune Cells in the Lungs

**Figure 5:**
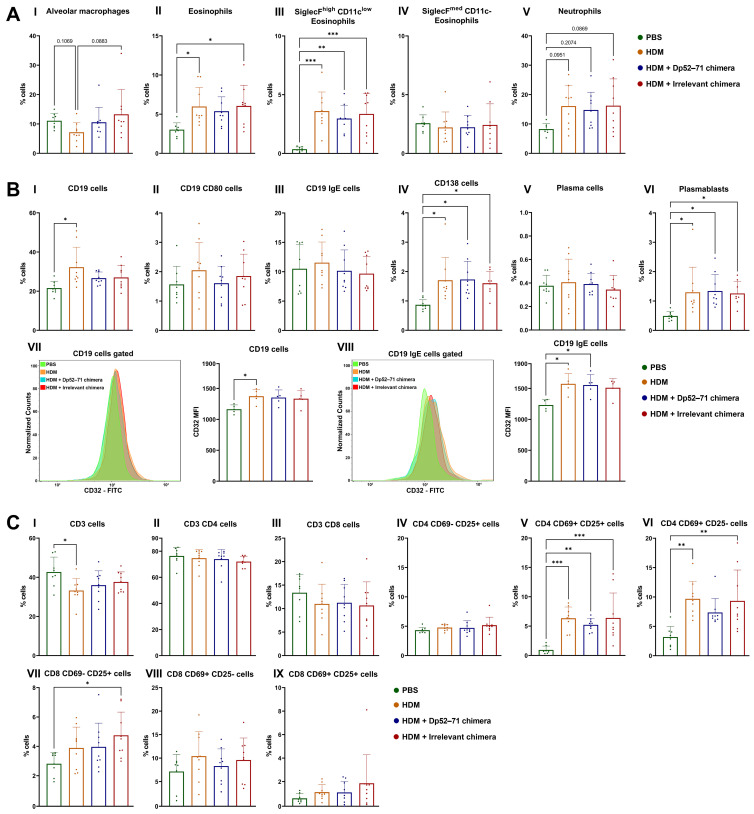
Phenotyping of immune cells in the lungs by FACS analyses. (**A**) Characterization of myeloid cell types—alveolar macrophages (**I**), SiglecF^high^ CD11c^low^ (**III**), SiglecF^med^ CD11c− (**IV**) and total eosinophils (**II**), and neutrophils (**V**)—presented as the percentage of the parent population. (**B**) Analysis of B and antibody-secreting cells—percentages of CD19 cells (**I**), CD19 CD80 cells (**II**), CD19 IgE cells (**III**), CD138 cells (**IV**), plasma cells (**V**), plasmablasts (**VI**), and CD32 mean fluorescent intensity (MFI) of CD19 (**VII**) and CD19 IgE cells (**VIII**). (**C**) Phenotyping of T cells—CD3 cells (**I**), CD3 CD4 cells (**II**), CD3 CD8 cells (**III**), CD4 CD69 − CD25 + cells (**IV**), CD4 CD69 + CD25 + cells (**V**), CD4 CD69 + CD25 − cells (**VI**), CD8 CD69 − CD25 + cells (**VII**), CD8 CD69 + CD25 − cells (**VIII**), and CD4 CD69 + CD25 + cells (**IX**). Data are shown as mean ± SD of 8–9 mice per group. The differences between the groups were evaluated using a one-way ANOVA followed by Tukey’s multiple comparisons, or a Kruskal–Wallis test followed by Dunn’s multiple comparisons test, depending on the normality of the data; *p* values are indicated on the graphs: * *p* < 0.05; ** *p* < 0.01; *** *p* < 0.001. Data are representative of at least 4 independent experiments.

We further analyzed the immune cell populations in the lungs to better characterize the ongoing inflammation (Supplementary Figure S1). First, we investigated the phenotype of the myeloid cells that are known to play a crucial role in this type of inflammation (Figure 5A). The percentage of eosinophils in the lungs was elevated in the HDM and the irrelevant chimera groups compared to the healthy controls (Figure 5(AII)). Two different populations of eosinophils were gated based on the surface markers SiglecF and CD11c. SiglecF^high^ CD11c^low^ eosinophils, described in the literature as a more activated population, were increased in the HDM-challenged groups in comparison to the healthy animals (Figure 5(AIII)). A non-significant downtrend in the percentage of these cells in the Dp52–71 chimera group was found, relative to the HDM group (*p* = 0.7483). There were no differences between the groups for the second distinguished eosinophilic population, characterized as SiglecF^med^ CD11c- (Figure 5(AIV)), and described as tissue-resident and less activated [33,34]. The populations of alveolar macrophages also did not differ between the groups (Figure 5(AI)), whereas a non-significant increase in neutrophils in all the HDM-challenged groups was found (Figure 5(AV)).

Since the chimeric molecules target allergen-specific B cells, characterization of this cell type, as well as of antibody-secreting cells, is of great interest to our research (Figure 5B). FACS analyses revealed a significantly increased number of CD19+ cells in the HDM group in comparison to the healthy mice (Figure 5(BI)). A non-significant increase was also observed in the Dp52–71 and the irrelevant chimera groups.

The expression of CD32 (FcγRIIb) on B cells is important as it is a target protein for our chimeric molecules. The expression (MFI) of CD32 on CD19 cells (Figure 5(BVII)), as well as on CD19 + IgE + cells (Figure 5(BVIII)), was significantly increased in the HDM-challenged group compared to the PBS-treated mice. In some cases, FcγRIIb can trigger inhibitory signals in B cells independently of the B cell receptor (BCR) [35,36]. This overexpression in HDM-challenged mice can explain the similar trends in some parameters shared between the Dp52–71 and the irrelevant chimera groups. The administration of Dp52–71or irrelevant chimera did not influence the expression of CD32 on CD19 + and on CD19 + IgE + cells when compared to the HDM-challenged mice.

The percentages of total antibody-secreting cells (CD138 positive cells) (Figure 5(BIV)) and plasmablasts (Figure 5(BVI)) were higher in all the HDM-challenged groups than in the healthy mice. No significant differences between the groups in terms of the number of plasma cells (Figure 5(BV)), or cells expressing CD19 + CD80 + (Figure 5(BII)) or CD19 + IgE + (Figure 5(BIII)), were found.

T cell subtypes in the lungs of the HDM-challenged mice were also analyzed (Figure 5C). This analysis showed a significantly decreased number of CD3 + cells in the HDM group compared to the healthy mice (Figure 5(CI)). However, the groups had no differences in the number of CD3 + CD4 + (Figure 5(CII)) and CD3 + CD8 + T cells (Figure 5(CIII)). The activated T cell subtypes CD4 + CD69 + CD25+ (Figure 5(CV)) and CD4 + CD69 + CD25− (Figure 5(CVI)) were increased in all the HDM-challenged groups compared to the control animals, whereas a non-significant reduction in these cells was found in the Dp52–71chimera group relative to the HDM-challenged mice without treatment (CD4 + CD69 + CD25 +, *p* = 0.7675; CD4 + CD69 + CD25−, *p* = 0.4809). Activated CD8 + subpopulations showed no significant differences between the groups (Figure 5(BVII,BVIII)).

### 2.6. Histopathological Examination of Mouse Lungs

**Figure 6:**
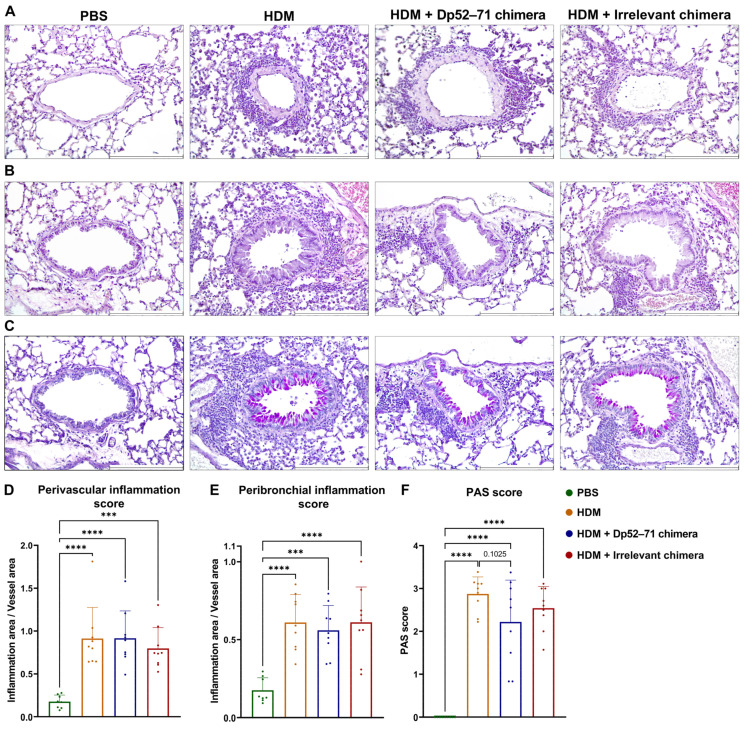
Histological analysis of lung pathology. Representative images and histological score of perivascular (**A**,**D**) and peribronchial (**B**,**E**) inflammation of H&E-stained lung tissue. Periodic acid-Schiff (PAS) score (**F**) and representative images for mucus production (**C**). Scale bars, 250 µm. Data are shown as mean ± SD of 8–9 mice per group. *p* values were calculated using a one-way ANOVA followed by Tukey’s multiple comparisons test; *p* values are indicated on the graphs: *** *p* < 0.001; **** *p* < 0.0001. Data are representative of at least 4 independent experiments.

Finally, the overall allergic-like inflammation in the lungs was studied by histological analysis. Hematoxylin and eosin (H&E) staining of murine lung tissue revealed a perivascular (Figure 6A,D) and peribronchial (Figure 6B,E) cellular infiltration in all the HDM-challenged groups, without significant differences between them.

PAS-positive airway epithelial cells, representing the goblet cells responsible for mucus production, were found in the airways of the HDM-challenged groups, but not in the healthy mice (Figure 6C,F). A non-significant decrease in histopathological PAS score was shown in the animals administered Dp52–71chimera compared to the HDM group (*p* = 0.1025).

### 2.7. Correlation Analysis of HDM-Challenged Mice

**Figure 7:**
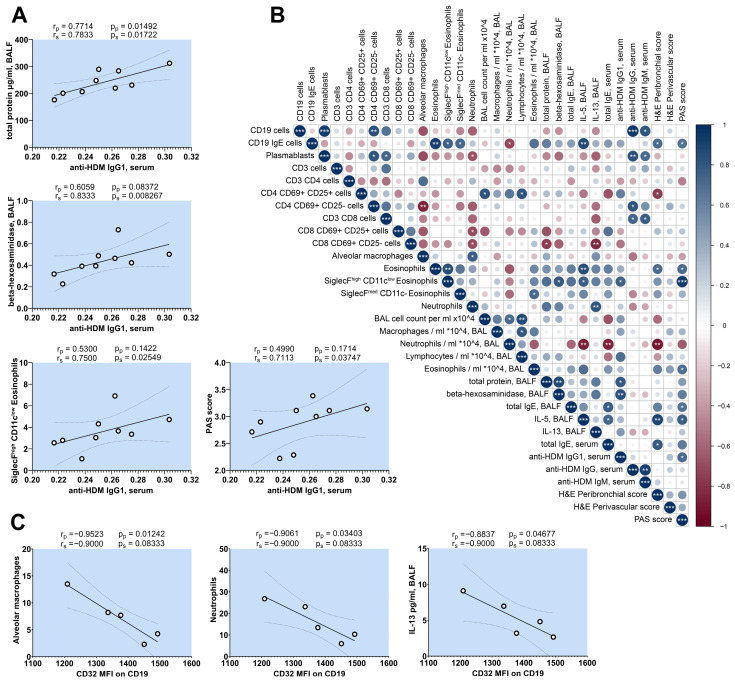
Correlation analysis of HDM allergy group. Correlation analysis of serum levels of anti-HDM IgG1 (**A**) and CD32 expression of B cells in the lungs (**C**) versus other parameters. Pearson (rp) and Spearman (rs) correlation coefficients and *p* values for Spearman (ps) and Pearson (pp) correlation analysis are indicated above each figure. (**B**) Correlation matrix of key immunological parameters achieved through Spearman’s test (* *p* < 0.05; ** *p* < 0.01; *** *p* < 0.001).

Although mouse models for generating HDM-allergy have similar protocols, the outcome of their allergic inflammation often differs [37]. To better characterize our HDM model and the relationships between the different parameters investigated, we performed a correlation analysis within the HDM group. This additional data exploration may help us to better understand the relationship between the immunological parameters of interest. Figure 2C shows that the administration of Dp52–71 chimera led to a reduction in the levels of HDM-specific IgG1 antibodies in the serum. However, the influence of the Dp52–71 chimera on the other immunological parameters had no or little effects reaching statistical significance. For this reason, we performed a correlation analysis to investigate how anti-HDM IgG1 antibodies are related to the parameters that indicate allergic inflammation. We found strong statistically significant positive correlations between anti-HDM IgG1 antibodies and total protein concentration in BALF, enzyme activity of β-hexosaminidase in BALF, percentages of SiglecF^high^ CD11c^low^ eosinophils in the lungs, and the PAS score of the lungs (Figure 7A). These results suggest that the non-statistically significant trends observed between the Dp52–71 chimeric group and the HDM group were not the result of just one measured point, but rather the result of the small effect generated by targeting the B cells with an epitope which is a part of only one allergen.

The number of SiglecF^high^ CD11c^low^ eosinophils in the lungs showed a positive correlations with β-hexosaminidase and IL-5 in BALF, and a strong positive correlation with the PAS score (Figure 7B). Interestingly, the serum levels of anti-HDM IgG antibodies showed no correlations with the parameters, correlated with anti-HDM IgG1 antibodies (Figure 7B).

Another key finding in this research was the overexpression of CD32 on the surface of lung B cells in the HDM allergy model. In addition, we aimed to determine whether this overexpression correlated with other investigated immunological parameters in this murine model. The results showed a significant negative correlation between the CD32 MFI of B cells in the lungs and the percentage of alveolar macrophages and neutrophils in the lungs, as well as the concentration of IL-13 in BALF (Figure 7C).

## 3. Discussion

The present study aimed to target allergen-specific B cells in a chronic HDM allergy model, using a protein-engineered chimeric molecule, capable of the simultaneous engagement of BCR and CD32, as a novel therapeutic approach. Chimeric molecules consisting of a monoclonal 2.4G2 antibody that recognizes FcγRIIb coupled with proven epitope-carrying peptides (Dp52–71), and control chimeras with the same antibody, were constructed. The newly constructed chimeras retained their capacity to bind to FcγRIIb on B cells, and were recognized by allergen-specific IgG1 antibodies. Administration of the Dp52–71 chimera resulted in decreased levels of HDM-specific antibodies in the serum of mice with chronic intranasal exposure to HDM extract. However, the reduction in antibody levels was partial, possibly due to the targeting of a limited epitope-specific B cell population, whereas the antibody repertoire was broad against the various allergens found in the HDM extract. Moreover, some insignificant trends of reduction in several parameters, including BALF β-hexosaminidase, SiglecF^high^ CD11c^low^ eosinophils, and reaching the maximum confidence level of the lung PAS score (*p* = 0.1025), were observed. A Dp52–71 chimera was constructed to target mature epitope-specific B cells by co-crosslinking the BCR and FcγRIIb, and triggering the phosphorylation of the ITIM domain of FcγRIIb. This phosphorylation leads to the binding of SH2-domain-containing inositol phosphatase, SHIP1, resulting in the inhibition of activating signals by dephosphorylation of BCR cytoplasmic domain. However, SHIP- and ITIM-independent signaling through FcγRIIb in the absence of a BCR trigger is capable of inducing apoptosis in mature B cells, by the activation of the pro-apoptotic BCL-2-antagonist of cell death (BAD) and BH3-interacting-domain death agonist (BID). Furthermore, the outcome of FcγRIIb ligation depends on the developmental stage of the B cells. While FcγRIIb co-crosslinking with BCR inhibits the proliferation of mature B cells and pro-B cells, FcγRIIb ligation alone leads to apoptosis of activated B cells, pre-B cells, and plasma cells [38,39]. Although the Dp52–71 chimera would have the ability to bind to untargeted cells to some extent, the chimera would bind preferentially to Dp52–71-specific B cells, because of the increased overall avidity equivalent to the sum of the binding affinities of the peptide to BCR and the 2.4G2 antibody to FcγRIIb. We included an irrelevant chimera group to investigate the effect of the 2.4G2 antibody alone, and to evaluate whether the limited binding to the untargeted cells affected their functionality in the HDM allergy model. There was no significant difference between the irrelevant chimera group and HDM group in the levels of HDM-specific IgG1. However, the lack of a statistically significant difference between the Dp52–71 and irrelevant chimera groups suggests that the limited binding to 2.4G2 of the irrelevant chimera could have an insignificant effect to some extent. This can be attributed to the alternative signaling cascades that could be activated independently of the BCR. Our previous studies explored the in vitro and in vivo effects of chimeric molecules that targeted the same Dp52–71-specific B cell population found in patients with HDM allergy through the inhibitory CR1 receptor expressed on human B cells. Treatment with these anti-human CR1 chimeric molecules containing the same epitope-bearing peptides as the mouse chimera reduced the number of anti-HDM IgE antibody-secreting cells, and induced B cell apoptosis in vitro [21]. The administration of an anti-human chimeric molecule in a humanized Rag2-γc-model of HDM allergy resulted in a reduction in HDM-specific IgE antibodies in both the serum and BALF. This treatment also reduced the total protein and β-hexosaminidase levels in BALF and immune cell infiltration in the lungs [22]. Although the use of both mouse and human chimeras resulted in a reduction in HDM-specific antibodies, the treatment outcomes for overall allergic disease differed, since the mouse chimera failed to reach a statistically significant reduction in other disease parameters. The reason for this difference may be due to several factors, such as the specificities of the targeted receptors, as well as the characteristics of humanized and mouse models. It is possible that the levels of the immune response against the immunodominant Der p1 allergen, particularly the Dp52–71 peptide, are different in the human allergy and the chronic mouse model of HDM allergy. Furthermore, the observed statistically insignificant trends between the Dp52–71 chimera and HDM groups matched with the parameters that showed a strong positive correlation with anti-HDM IgG1 antibodies (β-hexosaminidase in BALF, SiglecFhigh CD11clow eosinophils, and PAS score of the lungs). Along with these results, it is tempting to speculate that the reduction in HDM-specific IgG1 antibody levels can be associated with the trends that we observed between the Dp52–71 chimera and the HDM groups, and to attribute the lack of statistical significance for these parameters to the small size of the effect we generated by targeting B cells specific to only one allergen epitope.

The present study explored a new therapeutic strategy for targeting HDM allergen-specific B cells using chimeric molecules. A chronic mouse model of HDM allergy was developed to investigate the in vivo effects of the proposed therapy. This HDM allergy model was associated with elevated serum levels of HDM-specific IgG and IgG1 antibodies. However, no differences were observed in the total and HDM-specific IgE levels between the healthy and HDM-challenged mice. Some studies using mouse HDM allergy models have been able to induce HDM-specific IgE antibodies, but this increase was often very weak compared to that observed in HDM-allergic asthma patients [40]. Other authors who used murine models of HDM allergies focused on the analysis of HDM-specific IgG1 antibodies, and either measured no levels of anti-HDM IgE antibodies, or failed to detect elevated allergen-specific IgE levels [40,41,42,43,44]. Moreover, differences in the biochemical composition of HDM extracts, such as protease content, have been reported to be responsible for total and HDM-specific IgE responses, but not for anti-HDM IgG1 [37,45]. Furthermore, experiments involving FcεRIα- or IgE-deficient mice, in a short 14-day HDM allergy model with large or limited doses of allergens, have suggested that neither FcεRIα nor IgE contribute to allergic airway disease [46]. The lack of IgE response in our chronic mouse model of HDM allergy could be attributed to the biochemical composition of the HDM extract batch, or other factors such as the treatment schedule. However, the presence of a Th2-dependent HDM-specific IgG1 response, with increased pulmonary inflammation and mucus secretion, points towards a state of allergic asthma. It is also possible that the Dp52–71 chimera has the same or even greater effect in a model characterized by elevated allergen-specific IgE antibodies due to the overexpression of FcγRIIb found in IgE-expressing B cells and IgE-associated pathological mechanisms.

Animal models of HDM allergy are a useful tool for exploring disease pathogenesis and potential therapeutics. However, differences between allergic models, based on factors such as the biochemical composition of HDM extracts, alongside allergen administration protocols, warrant further in-depth analyses of the models. Here, we aimed to characterize our chronic model in detail by examining both humoral and cellular responses. The analyses revealed that HDM-challenged mice had increased numbers of eosinophils, neutrophils, and lymphocytes in the BAL fluid, as well as increased percentages of SiglecF^high^ CD11c^low^ eosinophils, CD19 cells, plasmablasts, and activated CD4 CD69 + CD25 + T cells in the lungs. Elevated levels of total protein in BALF, β-hexosaminidase activity, IL-5, and HDM-specific IgG1 and IgG were also found in the HDM group. The mouse model of HDM allergy was associated with histopathological changes in the lungs, such as peribronchial and perivascular inflammation and goblet cell hyperplasia. Correlation analysis of immunological parameters in the current model showed an intriguing relationship between serum levels of HDM-specific IgG1 and several key parameters of allergic inflammation. We found a strong positive correlation of HDM-specific IgG1 levels with total protein and β-hexosaminidase activity in BALF, SiglecF^high^ CD11c^low^ eosinophils in the lungs, and lung PAS score. Importantly, the serum levels of anti-HDM IgG antibodies showed no correlation with these parameters, suggesting that IgG1 subclass antibodies play a different role in allergic inflammation. Although many authors have described increased levels of HDM-specific IgG1 antibodies and used them as a marker of antigen-specific B and T cell clonal expansion, the contribution of this subclass of antibodies to allergic inflammation needs clarification, especially in regard to sensitization to HDM allergens [40]. However, experiments investigating the role of allergen-specific IgG1 in hypersensitivity reactions to ovalbumin have shed light on possible mechanisms. Mice passively sensitized with ovalbumin-specific IgG1 developed positive skin test reactivity and an increased airway responsiveness after airway challenge accompanied by increased BAL eosinophils. Furthermore, passive immunization with ovalbumin-specific IgG2a or IgG3 was not associated with these reactions [47].

Another study found that in mice, death and most of the pathophysiological changes associated with active or IgG1-dependent passive anaphylaxis depend on the FcR gamma chain. The authors also suggested that systemic anaphylaxis may be significantly mediated by FcγRIII and IgG1 [48]. Furthermore, FcγRIII-deficient mice have impaired IgG-mediated passive cutaneous anaphylaxis and mast cell degranulation [49]. Additionally, FcγR-mediated uptake of allergen-specific IgG immune complexes by dendritic cells may contribute to airway inflammation. The authors observed that i.n. administration of ovalbumin-specific IgG immune complexes leads to the enhancement of Th2 cytokine production, antigen-specific T cell proliferation, eosinophilia, and airway inflammation [50,51]. Elevated serum levels of HDM-specific IgG1 in our allergy model may have contributed to the positively correlated total protein and β-hexosaminidase activity in BALF, SiglecF^high^ CD11c^low^ eosinophils in the lungs, and lung PAS score, as the study focused on ovalbumin-specific IgG1, providing evidence for a role of IgG1 in hypersensitivity, including skin test reactivity, airway responsiveness, eosinophilia, mast cell degranulation, and anaphylaxis. Although it is possible that Th2-associated allergen-specific IgG1 may be involved in the development of several features of HDM allergy in mice, more studies are needed, as correlation analysis is not sufficient for definitive conclusions.

Examination of the expression of our target molecule CD32 (FcγRIIb) showed that this receptor was overexpressed on the surfaces of CD19 and CD19 IgE-positive B cells in the lungs of HDM-challenged mice, compared to the healthy animals. This overexpression was not affected by the administration of FcγRIIb-specific Dp52–71 or irrelevant chimeras. Furthermore, CD32 overexpression showed a statistically significant negative correlation with the percentages of alveolar macrophages and neutrophils in the lungs, as well as IL-13 concentration in BALF. To our knowledge, this is the first study to show the overexpression of FcγRIIb on B cells in HDM allergy. Maternal immunization with ovalbumin has been shown to result in the overexpression of FcγRIIb in the splenic IgM + B cells of offspring, in an IL-10-dependent manner [52,53]. Surprisingly, similar preconception immunization with *Dermatophagoides pteronyssinus* allergens resulted in the reduced expression of FcγRIIb on the B cells of offspring [54]. However, these findings cannot be directly compared with our results, based on several factors. First, our study demonstrated the direct effect of HDM extract on B cell expression of FcγRIIb, but not the effect of maternal immunization on offspring. In addition, we analyzed local lung B cells, and our allergen administration was intranasal, whereas spleen B cells and the systemic route of allergen administration were used in the aforementioned study. The role of FcγRIIb in allergic diseases is mainly highlighted in effector cell functions, such as the inhibition of IgE-induced mast cell activation [55,56,57]. However, the expression of FcγRIIb on B cells appeared to be critical for the development of mucosal antigen-induced tolerance, which was impaired in FcγRIIb−/− mice. The transfer of B cells expressing FcγRIIb is sufficient to induce Foxp3+ regulatory T cells and suppress antigen-specific T cells. Furthermore, FcγRIIb-overexpressing B cells have an increased capacity to induce mucosal T cell tolerance and regulatory T cells in B cell-null mice [58]. The HDM-induced overexpression of FcγRIIb on lung B cells in the chronic mouse model, and the negative correlation with neutrophil and IL-13 concentration, are intriguing. It is difficult to predict whether FcγRIIb expression on CD19 and CD19 IgE-positive B cells affects these parameters, or whether these associations have different origins based on correlation analysis. However, if IL-13 concentration and neutrophil counts are affected by the increased signaling capabilities of FcγRIIb due to the overexpression, this can provide an opportunity for future FcγRIIb-targeting strategies to be developed. Therefore, further studies are necessary to investigate the role of FcγRIIb on B cells in the context of allergic diseases, especially for HDM sensitization.

Non-sensitized mice were used under the same treatment regimen with both chimeras, to collect data on treatment safety and the absence of side effects. No statistically significant changes in the observed parameters and symptoms were found for the controls.

Here, we investigated a possible new approach for the specific therapy of HDM allergy, using protein-engineered molecules that lead to a reduction in HDM-specific antibody levels. A limitation of this study is that we cannot expect to suppress the entire spectrum of allergen-specific B cells in HDM allergy using only one Der p1 epitope; however, the suppression of B lymphocytes specific to this epitope provides the opportunity for future multi-epitope delivery. The selective elimination of allergen-specific B cells by chimeric molecules has promising translational potential, and can be further developed in a novel approach to specific therapy. The present study suggests the future use of FcγRIIb overexpression and the expansion of protein chimeric technology with more epitopes to improve the outcome of inflammation.

## 4. Materials and Methods

### 4.1. Antibodies

Rat 2.4G2 hybridoma, which produces a monoclonal IgG2b antibody specific to mouse FcγRII (CD32) (ATCC HB-197), was cultured in serum-free CHO medium (Gibco, Gaithersburg, MD, USA). The antibodies were isolated and purified from the supernatant as described above [29].

The following were used for the fluorescence-activated cell sorting (FACS) experiments: Fluorescein isothiocyanate (FITC)-conjugated anti-mouse CD16/32 antibody (Cat# 35-0161-U100, clone 2.4G2, Tonbo Bioscience, San Diego, CA, USA), anti-rat IgG-FITC antibody (Cat# 405404, BioLegend, San Diego, CA, USA), anti-mouse FITC-conjugated CD11c (Cat# 117306, clone N418, BioLegend, San Diego, CA, USA), CD25 (Cat# 102006, clone PC61, BioLegend, San Diego, CA, USA), and CD45R (B220) (Cat# 103206, clone RA3-6B2, BioLegend, San Diego, CA, USA); Phycoerythrin (PE)-conjugated CD19 (Cat# 115508, clone 6D5, BioLegend, San Diego, CA, USA), SiglecF (Cat# 12-1702-80, clone 1RNM44N, eBioscience, Frankfurt, Germany), CD69 (Cat# 104508, clone H1.2F3, BioLegend, San Diego, CA, USA), and CD138 (Cat# FAB2966P, clone 300506, R&D Systems, Minneapolis, MN, USA); PE-Cyanine 7 (PE-Cy7)–conjugated CD3 (Cat# 100320, clone 145-2C11, BioLegend, San Diego, CA, USA) and CD8 (Cat# 100722, clone 53-6.7, BioLegend, San Diego, CA, USA); Pacific Blue-conjugated Ly6G (Cat# 127612, clone 1A8, BioLegend, San Diego, CA, USA), CD3 (Cat# 100334, clone 145-2C11, BioLegend, San Diego, CA, USA), and CD80 (Cat# 104724, clone 16-10A1, BioLegend, San Diego, CA, USA); PE-Cyanine 5 (PE-Cy5)–conjugated CD4 (Cat# 100410, clone GK1.5, BioLegend, San Diego, CA, USA) and Streptavidin (Cat# 15-4317-82, eBioscience, San Diego, CA, USA); and biotin-conjugated IgE (Cat# 13-5992-85, clone 23G3, eBioscience, San Diego, CA, USA) mAbs. Relevant Isotype-matched control IgG antibodies were used for compensation and primary antibody staining validation. Horseradish peroxidase (HRP)-conjugated anti-mouse IgG (Cat# 405306, BioLegend, San Diego, CA, USA), biotin-conjugated anti-mouse IgG1 (Cat# 406604, clone RMG1-1, BioLegend, San Diego, CA, USA), IgM (Cat# 406504, clone RMM-1, BioLegend, San Diego, CA, USA), IgA (Cat# 407004, clone RMA-1, BioLegend, San Diego, CA, USA), IgE (Cat# 13-5992-82, clone 23G3, eBioscience, San Diego, CA, USA), and Streptavidin-conjugated HRP (Cat# S5512 Sigma-Aldrich, Taufkirchen, Germany) were used for the enzyme-linked immunosorbent (ELISA) assays.

### 4.2. Generation of Protein-Engineered Chimeric Antibody Molecules

Two synthetic peptides and an mAb 2.4G2 specific to mouse CD32 were used for the construction of protein-engineered chimeric molecules. Selected epitope-bearing Dp52–71 peptides from Der p 1 (Ac- NQSLDLAEQELVDCASQHGC-Ahx-K-NH_2_), and irrelevant peptides (Ac- DEACLQCGSEDHQAVQNLLS-Ahx-K-NH_2_), consisting of the same amino acids in random order, were purchased from Caslo Laboratory (Lyngby, Denmark). During the peptide synthesis, using Fmoc chemistry on a resin with >96% purity, an Ahx linker with lysine was introduced to the peptides’ C-end. The Dp52–71chimera was constructed by coupling the 2.4G2 antibody with Der p1 peptides. An irrelevant chimera, consisting of the same antibody conjugated to irrelevant peptides, was also generated. The protein engineering chemical conjugation was performed as previously described [59]. Briefly, the classical EDC (1-ethyl-3(3′-dimethylaminopropyl) carbodiimide.HCl) (Cat# E6383, Sigma-Aldrich, Burlington, MA, USA) crosslinking technique was applied to bind the peptides at a 20-fold molar excess to the 2.4G2 antibody, with a 60-fold molar excess of the zero-length crosslinking agent carbodiimide. The protein–peptides reaction mixture was stirred overnight at 4 °C, followed by dialysis against PBS and concentration by ultrafiltration (Amicon XM50 membranes; Merck, Darmstadt, Germany).

### 4.3. Characterization of the Chimeric Molecules

#### 4.3.1. Recognition of FcγRIIb by Chimeric Molecules

The spleens from the healthy and HDM-challenged mice were taken, and a splenocyte single cell suspension was obtained by passing them through 70 µm cell strainer. Following erythrocyte lysis in hypotonic buffer, the splenocytes were washed and distributed into FACS tubes (2 × 10^5^ cells/tube) for further analysis.

The binding of the chimeric molecules to B and T cells was investigated by incubation of the spleen cells with 2.4G2 antibody, Dp52–71 chimera, or irrelevant chimera (0.8 µg/tube) for 20 min on ice, followed by detection with anti-rat IgG-FITC. Next, the splenocytes were washed with FACS buffer [2.5% fetal calf serum (FCS) in PBS] and incubated with CD3-PE-Cy7 and CD19-PE.

For another competitive analysis, the splenocytes were incubated with serial dilutions of mAb 2.4G2, Dp52–71 chimera, or irrelevant chimera (0.2, 0.4, 0.8, 1.6 µg/tube) on ice for 20 min. After washing, an antibody mixture containing anti-CD16/CD32 antibody-FITC, CD3-PE-Cy7, and CD19-PE antibodies was added.

A total of 30,000 cells in the lymphocyte gate were acquired using a BD LSR II flow cytometer (BD Biosciences, Mountain View, CA, USA), and analyzed using FlowJo software v10.10.0.

#### 4.3.2. Chimeric Molecule Recognition by Serum IgG1 Antibodies

Dp52–71 or irrelevant chimeras (100 µL, 10 µg/mL) diluted in coating buffer (0.015M Na_2_CO_3_, 0.035M NaHCO_3_, pH 9.6) were loaded onto a 96-well plate and incubated overnight at 4 °C. The wells were blocked with 200 µL 1% bovine serum albumin (BSA) in 0.05% Tween 20 in PBS (T-PBS) for 2h at room temperature (RT). The plates were incubated with serial dilutions (10×, 30×, 90×, 270×) of sera from healthy BALB/c mice, as well as from animals, with four subcutaneous injections (days 0, 21, 35, 49) with 100 μg/mouse of HDM allergen extract (Citeq biologics, Groningen, The Netherlands), adsorbed on 100 µg Al(OH)_3_ (Alum) for 2 h at RT. Then, biotin-conjugated anti-mouse IgG1 (1000× diluted) was added for 1 h of incubation at RT, followed by incubation with Streptavidin-conjugated HRP. After extensive washing, the enzyme reaction was developed with 0.1 mg/mL 3,3′,5,5′-tetramethylbenzidine (TMB) substrate (100 µL/well, Cat# 87748, Sigma-Aldrich, Burlington, MA, USA) in 0.05 M Phosphate-Citrate Buffer (pH 5.0) and 1.96 mM H_2_O_2_. The reaction was stopped by adding 2N H_2_SO_4_ (50 µL/well), and measured at 450 nm with correction at 570 nm (CLARIOstar Plus, BMG LABTECH, Ortenberg, Germany).

### 4.4. Mice

Female, 8-week-old BALB/c mice were purchased from the Jackson Laboratory (Bar Harbor, ME, USA) and housed in a specific pathogen-free (SPF) animal facility at the Institute of Microbiology. The experiments were conducted in strict adherence to the guidelines for the Animal Care Commission at the Bulgarian Food Safety Agency (BFSA), in accordance with the Care and Use of Laboratory Animals of the European Union (EU Directive 2010/63/EU), and in compliance with national regulations.

### 4.5. Generation of Mouse HDM Allergy Model and Treatment Schedule

The BALB/c mice were randomized and divided in 4 groups (9 animals per group). The sample size was determined based on data from previously published reports, rather than statistical considerations. The mice were anesthetized, and 25 µg HDM allergen extract in 35 µL PBS was administered intranasally (i.n.) in three of the groups. An HDM extract challenge was performed twice a week (with a one-day interval between challenges) for two weeks (days 0, 2, 7 and 9), followed by five additional administrations once a week (days 14, 22, 29, 36, and 43). For the last four weeks, the mice were injected intravenously (i.v.) once a week with either 50 µg Dp52–71 chimera, irrelevant chimera, or PBS, in doses of 100 µL a day, before the i.n. challenge (days 21, 28, 35, 42). The mice were bled from the retro-orbital sinus 24 h after the last HDM allergen challenge, and the collected mouse sera were kept frozen at −80 °C. The animals were sacrificed, and their bronchoalveolar lavage fluid (BALF), lungs, and spleens were collected. The control mouse group was treated with PBS alone in the same manner.

### 4.6. BALF Collection and Differential Cell Count

Three volumes of 1 mL ice-cold PBS were introduced into the mouse lungs, and the recovered liquid volume was measured. The samples were centrifuged for 10 min at 600× *g* at 4 °C, and the supernatant of the first injection volume was collected and stored at −80 °C for subsequent BALF analysis. The cell pellets from the three lung washing volumes for each mouse were pooled and centrifuged again. The erythrocytes were then lysed with 200 µL lysis buffer, followed by centrifugation. The pellets were reconstituted in 500 µL PBS, and the cells were counted with a hemocytometer. Fifty thousand cells were placed on cytospin slides and centrifuged for 10 min at 700 rpm. The air-dried slides were stained with a Differential Quik Stain Kit (Cat# 24606, Polysciences, Niles, IL, USA), according to the manufacturer’s instructions. The slides were observed using a microscope (200× magnification, Leica DM2000, Leica Microsystems, Wetzlar, Germany), and six fields per slide were analyzed by ImageJ software v 1.54g (200–400 cells per mouse). Finally, the cells were calculated as cells per mL recovered liquid and percentages.

### 4.7. Detection of Anti-HDM IgG, IgG1, IgM, IgA, and IgE in Sera and BALF

HDM allergen extract (10 µg/mL in Coating buffer, pH 9.6) was loaded onto a 96-well plate (50 µL/well) and incubated overnight at 4 °C. The wells were washed with T-PBS and blocked with 200 µL/well 1% BSA in T-PBS for 2h at RT. Then, 50× diluted mouse sera, or 3× diluted BALFs in 1% BSA in T-PBS (50 µL per well), were added and incubated for 2h at RT. The plates were extensively washed and secondary incubated with 2000× diluted HRP-conjugated anti-mouse IgG (50 µL/well), or with 1000× diluted biotin-conjugated anti-mouse IgG1, IgM, or IgA, for 1 h at RT. The samples with biotin conjugates were incubated for an additional 1 h with 500× diluted streptavidin-conjugated HRP. The enzyme reaction was developed by adding TMB substrate as described above. The limit of detection was estimated as 3 times the standard deviation (SD) of the blank samples, and then the mean optical density (OD) of the blank wells was subtracted from the OD of the serum samples [60].

HDM-specific IgE antibodies were examined by high-sensitivity fluorescence ELISA, using opaque 96-well plates and a similar protocol as TMB ELISA, with the following differences. The plates were coated with 50 µL of 50 µg/mL HDM; the sera were diluted 20× and incubated overnight at 4 °C; and the enzyme reaction was developed by adding 100 µM Amplex Red (Cat# A12222, Invitrogen, Waltham, MA, USA) and 2 mM H_2_O_2_ in 0.05 M sodium phosphate buffer (pH 7.4).

### 4.8. Assessment of Total IgE Antibodies, IL-4, IL-5, IL-9, and IL-13

The concentration of total IgE antibodies was investigated in BALF (10× diluted) and sera (800× diluted) with the ELISA MAX™ Deluxe Set, following the manufacturer’s instructions (Cat# 432404, Bioligand, San Diego, CA, USA).

The concentrations of IL-4 (Cat# 431104, BioLegend, San Diego, CA, USA), IL-5 (Cat# 431204, BioLegend, San Diego, CA, USA), IL-9 (Cat# 88-8092, Invitrogen, Waltham, MA, USA), and IL-13 (Cat# 88-7137, Invitrogen, Waltham, MA, USA) in BALF were examined using the manufacturer’s protocol with slight modifications. The volumes of the coating antibody, BALF samples (2.15× diluted), detection antibody, and enzyme substrate were modified to 50 µL/well.

### 4.9. Total Protein and β-Hexosaminidase Activity Measurements in BALF

The enzyme activity of β-hexosaminidase in the BALF samples, used as a marker of mast cell degranulation in the lungs, was analyzed as previously described [22]. Briefly, 50 μL of 5 mM p-nitrophenyl N-acetyl-b-d-glucosaminide (Cat# 4062.2, Carl Roth, Karlsruhe, Germany) in 50 mM trisodium citrate buffer, pH 4.5, (Cat# S1804, Sigma-Aldrich, Burlington, MA, USA) was incubated with 50 μL of BALF for 2 h at 37 °C. The reaction was stopped by adding 200 μL of 0.2 M glycine-NaOH, pH 10.6 (Cat# 15527, Riedel-de-Haën, Charlotte, NC, USA; Cat# S5881, Sigma-Aldrich), and absorbance was measured at 405 nm.

The total protein concentration in the BALF was evaluated using Bradford reagent (Cat# B6916, Sigma-Aldrich, Burlington, MA, USA), according to the manufacturer’s instructions.

### 4.10. Phenotyping of Lung Infiltrates

The right lung lobes were cut into small pieces and incubated with digestion buffer containing 0.1 mg/mL DNase I (Cat# 10104159001, Sigma-Aldrich) and 2 mg/mL collagenase D (Cat# 11088858001, Sigma-Aldrich) for 45 min at 37 °C. A single cell suspension was prepared by passing the lungs through a 70 µm cell strainer. The cells were counted, placed in FACS tubes (2 × 10^5^/tube), and washed twice with FACS buffer. Phenotyping of the immune cells was achieved by incubation with the following four anti-mouse antibody cocktails for 20 min on ice: myeloid cells with gating-Ly6G-Pacific Blue, CD11c-FITC, and SiglecF-PE; B cells with gating-CD19-PE, CD80-Pacific blue, CD16/CD32-FITC, and IgE-biotin, and a second incubation with Streptavidin-PE-Cy5; antibody-secreting cells with gating-CD138-PE and CD45R (B220)-FITC; and T cells with gating-CD3-Pacific blue, CD4-PE-Cy5, CD8-PE-Cy7, CD25-FITC, and CD69-PE. The tubes were then washed twice and 30,000 cells were analyzed from each sample with a BD LSR II flow cytometer, using FlowJo software v10.10.0.

### 4.11. Histology

The left lung lobes from each mouse were fixed in 10% neutral buffered formalin (Cat# 3800598, Leica Biosystems, Wetzlar, Germany). The fixed tissue was processed (Leica TP1020, Leica Biosystems, Wetzlar, Germany) in increasing concentrations of ethanol (70%, 80%, 95%, and 100%) and Waxsol Cleaning Solution (Cat# S26.0390, Leica Biosystems, Wetzlar, Germany). The lungs were then embedded (Leica HistoCore Arcadia H and C, Leica Biosystems) in paraffin (Cat# P3558, Paraplast, Sigma-Aldrich, Wetzlar, Germany) and sectioned at 5 μm slices (Leica RM2125 RTS, Leica Biosystems, Wetzlar, Germany). The paraffin sections were analyzed by hematoxylin/eosin (Cat# 1.05175; #1.09844, Sigma-Aldrich) and Periodic acid-Schiff (Cat# PAS5-K-100, Biognost, Zagreb, Croatia) (PAS) staining. Scores for perivascular and peribronchial inflammation were calculated using the following formula: area of cellular infiltrates around vessels/vessel area [22]. The area was measured by ImageJ software v 1.54g, and a five-point grading system was used for the PAS score: 0, <0.5% PAS-positive cells; 1, <25%; 2, 25–50%; 3, 50–75%; and 4, >75% [61]. Six to thirteen bronchioles per mouse were analyzed.

### 4.12. Statistical Analysis

Most of the statistical analyses were performed with GraphPad Prism 9 software. The reported data were represented as mean ± SD, and a value of *p* < 0.05 was considered as statistically significant. The ELISA and cytokine samples were duplicated. The data normality was analyzed with Shapiro–Wilk and Kolmogorov–Smirnov tests. According to the data normality, statistical significance was assessed by a one-way or two-way ANOVA, followed by Tukey’s multiple comparisons, or a Kruskal–Wallis test continued by Dunn’s multiple comparisons test. Correlation analyses were performed using Spearman’s and Pearson’s tests. A Spearman’s correlation matrix was generated using SRplot on bioinformatics.com.cn [62].

## Data Availability

The data presented in this study are available on request from the corresponding author.

## Supplementary Materials

The following supporting information can be downloaded at https://www.mdpi.com/article/10.3390/ijms252413661/s1.
