# Supplementary material for: Suppression of Pathological Allergen-Specific B Cells by Protein-Engineered Molecules in a Mouse Model of Chronic House Dust Mite Allergy
Source: Int J Mol Sci. 2024 Dec 20;25(24):13661. doi: 10.3390/ijms252413661 (PMC11728213; doi:10.3390/ijms252413661)
Supplement: Supplementary file 1 [file ijms-25-13661-s001.zip › ijms-3340004-supplementary.pdf]

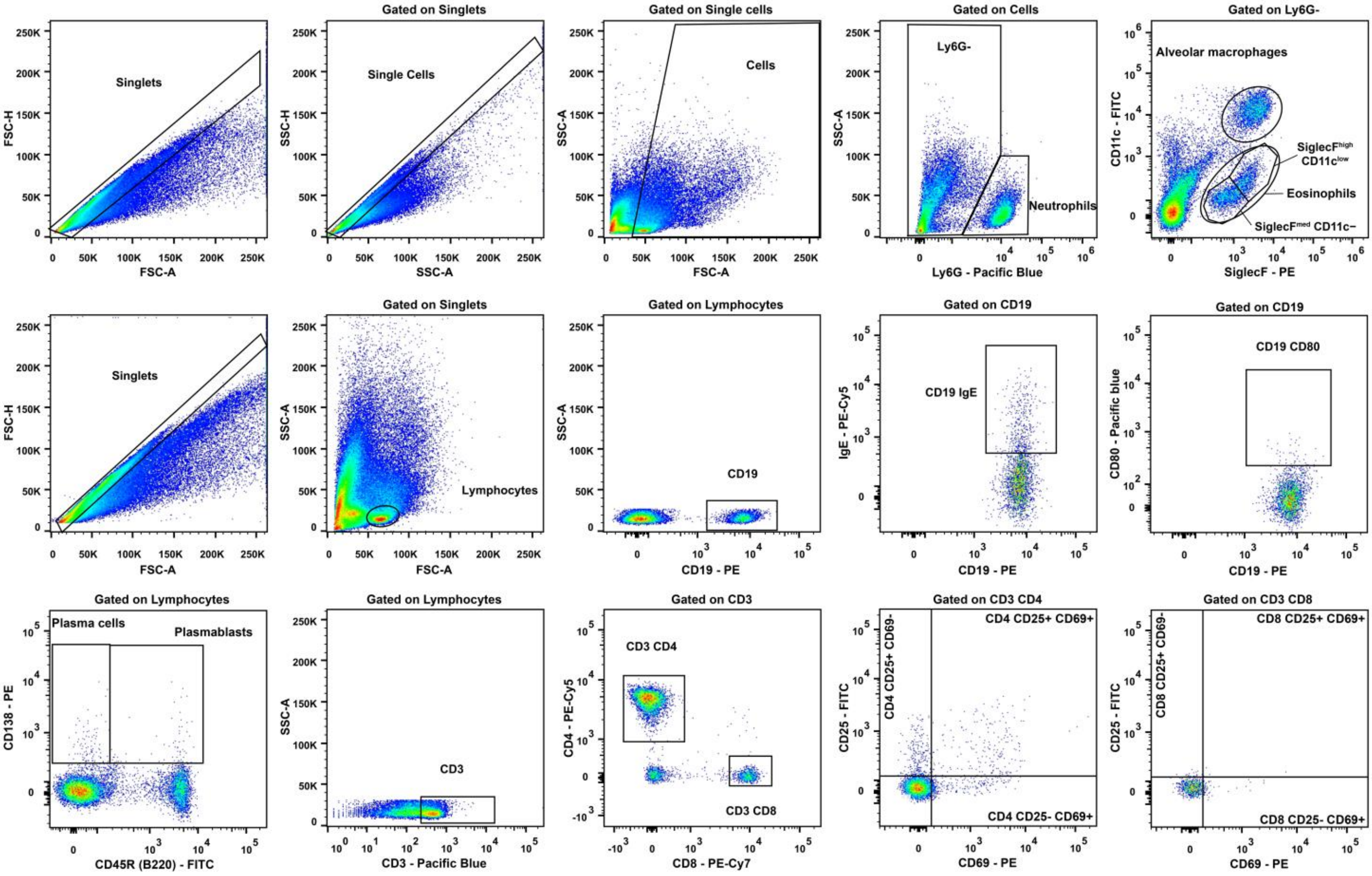


**Figure S1.** Gating strategy for phenotyping of immune cells in the lungs by FACS analysis. Gating strategy of myeloid cells (first row), B and antibody-secreting cells (second row), and T cells (third row).
